# News and Coming Events

**Published:** 1908-06-06

**Authors:** 


### News and Coming Events.

Dr. A. F. Hertz has been appointed to the Lectureship on Materia Medica and Pharmacology at the University of Oxford.

The President of the Board of Education has appointed Miss Janet M. Campbell, M.D., to the Medical Department of the Board.

Mr. Comyns Berkeley, B.A., M.B., B.C.(Cantab.), M.R.C.P., has been elected Obstetric Physician, and Mr. W. F. Victor Bonney, M.S., M.D., B.Sc., F.R.C.S., Assistant Obstetric Physician, to the Middlesex Hospital.

The death occurred at Launceston, Cornwall, on May 27, at the age of 76, of Dr. David Thompson, who had been coroner for North-East Cornwall for the past 19 years. Dr. Thompson had been Mayor of Launceston. He was an enthusiastic volunteer, having been gazetted hon. assistant surgeon in 1860, and he subsequently acted as brigade surgeon colonel. Dr. Thompson was a Justice of the Peace for the borough and county.

In Lord Mount Stephen's letter to the Prince of Wales which accompanied his last munificent donation to King Edward's Hospital Fund he expressed the hope that a few of the friends of the fund would unite in raising the further capital sum (about £300,000) required to increase its income from investments to £75,000 a year. His Royal Highness has received from Lord Revelstoke a gift of £500 as a contribution to the object thus suggested by Lord Mount Stephen.

The third annual meeting of the Convalescent Homes' Association was held at 32 Sackville Street, W., on May 28, Sir William S. Church, Bart., K.C.B., in the chair. A report was submitted regarding the provision that has been made at convalescent homes, at the instance of the Association, for patients requiring skilled attention and nursing after operation, and the use that has been made of the special beds; and the object and work of the Association was explained.

The annual meeting of the North India School of Medicine for Christian Women at Ludhaiana was held on May 27 at the Holborn Restaurant, Surgeon-General A. F. Bradshaw, C.B., Honorary Physician to the King, presiding. The objects of the school, which was founded in 1894, are to train Indian Christian women as medical missionaries to work in connection with Protestant Missionary Societies; to enable them to obtain a Government diploma in Medicine, Surgery, and Obstetrics; to train nurses, compounders, and midwives; and to train them all in evangelistic work among the patients of the Memorial Hospital, Ludhiana. Dr. D. N. P. Datta, a member of the local committee, spoke from experience of what was taking place on the spot. Colonel T. H. Hendley, C.I.E. (Inspector-General of Civil Hospitals, Bengal, 1898-1903), endorsed what the Chairman had said about the medical value of the school, and what Dr. Datta had said regarding its medical and evangelical value. Mr. W. Coldstream, I.C.S. (Retired) said that there was an urgent need for three ladies at once, and, in fact, there was a grave danger of the withdrawal of the Government grant if such ladies were not shortly appointed. They needed a qualified lady, as Lecturer on Medicine, to hold the appointment also of physician to the hospital; a qualified lady as lecturer on anatomy and bacteriologist to the hospital; and an educated lady for the post of house-mother to the school.

The annual meeting of the Friedenheim Hospital (Home of Peace for the Dying) will be held on Saturday, June 13th, at 3.15 p.m., in the Hampstead Conservatoire, Eton Avenue, Swiss Cottage, N.W. Her Highness Princess Louise Augusta of Schleswig-Holstein has consented to preside, and will be supported by Her Grace the Duchess of Somerset, John Langton, Esq., F.R.C.S. (Chairman), A. Pearce Gould, Esq., M.S., F.R.C.S., Percy J. F. Lush, Esq., M.B., M.R.C.S., A. T. Schofield, Esq., M.D., M.R.C.S. and others.

At the second sitting of the General Medical Council on Wednesday, May 27, Principal Donald MacAlister, having vacated the chair, was introduced as the representative of the University of Glasgow. He was then unanimously and with acclamation re-elected President of the Council. The remainder of the Council's sittings during last week and the early part of this week were chiefly devoted to the case of Mr. John P. Nicolas, M.R.C.S., L.R.C.P., of Catford, which had been adjourned from the last session. The decision of the Council in this case had not been given at the time of going to press with this issue.

At a meeting of the Council of the Hampstead General Hospital (with which is amalgamated the North-West London Hospital), held this week, it was agreed that the regular out-patient department at the hospital should be closed and any necessitous cases be referred to the separate department at Kentish Town. This is in accordance with the statement made in the Council's report for the past year, to the effect that under the recommendations of the Committee of Subscribers which reported on the proposal for the reconstitution of the medical staff and the amalgamation scheme, it was intended when practicable to discontinue the out-patient department, so far as the regular attendance of ordinary cases was concerned. The out-patient work at the Hampstead Hospital will therefore be confined to casualty cases.

At the Court of Governors of the Middlesex Hospital, held on May 28 under the chairmanship of Field-Marshal Lord Grenfell, Lord Cheylesmore referred to the fact that observation wards were being opened that day in the out-patient department for patients suffering from injuries not sufficiently severe to detain them in the hospital. Mr. Pearce Gould, senior surgeon to the hospital, seconded the adoption of the report, and, referring to the re-establishment of lying-in wards, said that this was a demonstration of the advance which had been made in medical science since the time when these wards were considered too dangerous for a general hospital. Much had been achieved by anxious, persistent, and devoted investigation in the laboratory which had revolutionised the whole practice in regard to midwifery. They might have done nothing yet as regarded cancer to equal the discoveries of Pasteur, Lister, and others in regard to infectious diseases, but what they had done in other fields could only be arrived at in this by the same methods of laborious and consistent research. By this time they were assured that light would come, and that there might be as great a revolution with regard to cancer as there had been with regard to puerperal fever. A great deal was now known; in many cases effectual prevention had been accomplished, while in others distinct benefit had followed modern treatment. Patients afflicted with cancer had attended the electrical and light department on numerous occasions, and had obtained alleviation of their suffering.

Sir Frederick Treves will take the chair at a dinner to be held at the Hôtel Cecil on Thursday, June 25, in celebration of the jubilee of the Royal Dental Hospital, Leicester Square. He makes a strong appeal for funds.

The Prince and Princess of Wales and Princess Christian have granted their patronage to the *matinée* which will be given at the Playhouse, lent by Mr. Cyril Maude, on Tuesday afternoon, June 23, at 3 o'clock, in aid of the Seamen's Hospital Society, *Dreadnought*, Greenwich. Three one-act plays will be presented.

On Tuesday, June 16, at an afternoon meeting at 11 Chandos Street, Cavendish Square, of the Society for the Study of Inebriety, Robert J. Parr, Esq., Director of the National Society for the Prevention of Cruelty to Children, will open a discussion on "Alcoholism and Cruelty to Children." The meeting will begin at 3.45 p.m.

Under the terms of the will of the late Mr. Edmund Richardson, the following institutions each receive a sum of 200 guineas: Charing Cross Hospital, the London Hospital, Middlesex Hospital, Brompton Cancer Hospital, University College Hospital, the Consumption Hospital, Fulham Road, the Royal Hospital for Incurables, the North-West London Hospital, the Royal Free Hospital, the Hospital for Sick Children, Great Ormond Street, and the Royal National Orthopædic Hospital.

A Dreschfeld memorial entrance scholarship in medicine has been established at the University of Manchester. It will be of the value of £30, tenable for one year, and will be awarded on the results of the matriculation examination in July of each year, the Joint Matriculation Board being asked to report on the work of candidates. Unless they have previously satisfied the requirements of the General Medical Council, candidates are required to do so. Successful candidates are required to enter for the first M.B. course or for the first year of the course of the conjoint colleges. The scholarship is to be open to both men and women candidates.

The Duke of Fife took the chair at the annual Court of Governors of the Hospital for Sick Children, Great Ormond Street. He said that there had been a gratifying increase in the number of students from the Colonies, showing that the hospital was becoming recognised as the chief teaching school in the Empire for children's diseases. The finances of the hospital caused great anxiety. There was a debt of £15,000, and the income was £6,000 a year below expenditure. He announced that the Queen had agreed to come in June to open the new out-patients' department. The report and accounts were adopted and the officers were elected.

On June 2, in the House of Commons, Captain Faber asked the Prime Minister whether, with reference to the meeting held in Berlin on May 23 with the object of founding an international association for the prosecution of cancer research, he would promise the assistance of this country in that direction. Mr. Asquith replied in the negative, stating that as at present advised he was not disposed to give such a promise as was suggested, and that the Imperial Cancer Research Fund, which is supported by Government, is doing all it can in the encouragement of individual intercourse and exchange of material between investigators of different countries.

The Post-Graduate College and Past and Present West London Hospital dinner will be held on Tuesday, June 23, at 7 for 7.30 p.m., at the Empire Rooms Trocadero Restaurant. Mr. L. A. Bidwell, F.R.C.S., will be in the chair.

The Council and Senate of Liverpool University have resolved to confer the honorary degree of Doctor of Law on Principal Donald Macalister. The degree will be conferred at the annual graduation ceremony on July 11.

Lord Rayleigh will visit Cambridge on Tuesday, June 16, Wednesday, June 17, and Thursday, June 18, in order to be installed as Chancellor. At four o'clock on Tuesday, June 16, he will open the new extension of the Cavendish Laboratory.

